# Trends in sexually transmitted and blood-borne infections in China from 2005 to 2021: a joinpoint regression model

**DOI:** 10.1186/s12879-023-08733-8

**Published:** 2023-10-30

**Authors:** Shuyuan Wang, Jialu Chen, Yuansheng Li, Beibei Zhang, Xiang Li, Ying Han, Junhui Zhang

**Affiliations:** Department of Epidemiology and Health Statistics, School of Public Health, Southwest Medical University, Longmatan District, No.1, Section 1, Xianglin Road, Luzhou, Sichuan People’s Republic of China

**Keywords:** Sexually transmitted and blood-borne infections, Joinpoint regression model, Diagnosis rate, Trend, China

## Abstract

**Background:**

Sexually transmitted and blood-borne infections (STBBIs) is a major public health concern in China. This study assessed the overall trends in STBBIs to improve the comprehensive understanding of the burden of STBBIs and provide evidence for their prevention and control.

**Methods:**

Data for the period from 2005 to 2021 were analyzed across China on infections with hepatitis B or C; syphilis; gonorrhea; and HIV infection. Trends, annual percent change (APC), and average annual percent change (AAPC) in diagnosis rate was analyzed using joinpoint regression models for the five STBBIs together or individually.

**Results:**

From 2005 to 2021, the overall diagnosis rate of all five STBBIs increased, with an AAPC of 1.3% [95% confidence interval (CI) -0.5% to 3.1%]. Diagnosis rates of HIV, syphilis and hepatitis C increased individually, but it decreased for infections of hepatitis B and gonorrhea. Joinpoint analysis identified four phases in diagnosis rate of hepatitis C; three phases in diagnosis rate of hepatitis B, HIV infection, and syphilis; two in diagnosis rate of gonorrhea infection.

**Conclusion:**

Despite national efforts to prevent and control STBBIs, their overall diagnosis rate has continued to rise in China, and they remain an important public health challenge. Further efforts should be made to educate the general population about STBBIs, particularly HIV. Interventions targeting vulnerable groups should be adopted and their efficacy monitored through regular analysis of trends.

**Supplementary Information:**

The online version contains supplementary material available at 10.1186/s12879-023-08733-8.

## Background

Sexually transmitted and blood-borne infections (STBBIs) are the most common infections around the world, especially in regions such as sub-Saharan Africa, Southeast Asia, and Latin America [1]. Common STBBIs include infections with hepatitis B or C, gonorrhea, syphilis and human immunodeficiency virus (HIV) [2]. Among them, HIV, syphilis, and gonorrhea are primarily transmitted through sexual contact, while hepatitis B and hepatitis C are mainly transmitted through blood [3]. In addition to causing physical and psychological harm to individuals, STBBIs also have significant social and economic consequences. In recent years, with globalization of the economy and social advances, incidence rates of STBBIs continue to rise [1, 4]. According to the World Health Organization in 2022, viral hepatitis and sexually transmitted infections collectively resulted in 2.3 million deaths annually, and each year, HIV, hepatitis B, or hepatitis C infected 4.5 million individuals. Although many governments have launched major initiatives to prevent and control STBBIs [5, 6], they remain a major, pressing public health problem worldwide [7].

Over the past thirty years, China has faced significant challenges in preventing and controlling STBBIs, as factors such as the acceleration of urbanization, frequent population mobility, and changes in sexual attitudes within the country have resulted in high incidence rates of these infections [8]. To address this situation, the Chinese government issued a series of policies to strengthen the prevention and control of STBBIs nationwide. For example, since the promulgation of the Infectious Diseases Prevention and Control Law in 1989 [9], syphilis and gonorrhea have been listed as legally notifiable infectious diseases; since 2002, HBV vaccination has been mandated for all newborns [10]; Regulations on the Prevention and Control of Sexually Transmitted Infections were enacted in 2012 [11]; and Guidelines for the Prevention and Control of Hepatitis C (Year 2019 Edition) were released in 2019 [12]. Under the guidance of these policies, together with comprehensive measures, certain achievements have been made in terms of STBBIs prevention and control [8]. However, the combined diagnosis rate of STBBIs in 2021 ranked first among the four categories of infectious diseases classified by the Chinese Center for Disease Control and Prevention (Chinese CDC) according to their transmission routes [13]. STBBIs remain a significant public health concern in China, causing enormous disease burden. Therefore, it is necessary to evaluate the trends of STBBIs in China in order to formulate more effective prevention and control measures and reduce the harm of STBBIs to public health.

Many studies have evaluated trends in STBBIs, finding that STBBIs are a serious public health problem in China. However, most of these studies used general descriptive epidemiological methods, which provided only summary information and lacked in-depth analysis of trends [14]. In contrast, joinpoint regression model can identify inflection points in data, calculate indicators such as annual percent change (APC) and average annual percent change (AAPC), and provide trend information for each inflection point, helping to better understand change trends [15]. Some studies have used this model to investigate either one (such as HIV infection [16], hepatitis B [17] and gonorrhea [18]), two (syphilis and gonorrhea [19], hepatitis B and hepatitis C [20]) or three (HIV infection, syphilis and gonorrhea [21]) STBBIs. These studies assessed the trends in certain STBBIs. However, there is a lack of literature analyzing the overall trends of STBBIs. Therefore, a comprehensive study analyzing STBBIs in China using the joinpoint regression model is necessary to understand the overall trend.

To address these gaps in knowledge, this study used joinpoint regression models to assess the overall trends in five STBBIs (HIV infection, hepatitis B, hepatitis C, syphilis, and gonorrhea) nationwide in China from 2005 to 2021, aiming to improve the comprehensive understanding of the burden of STBBIs and provide evidence for their prevention and control.

## Methods

### Data source

In China, infectious diseases are classified based on their severity into Class A, Class B, and Class C, totaling 40 diseases (including COVID-19) [22]. The Chinese CDC divides Class A and Class B infectious diseases into four types according to the routes of transmission: intestinal infectious diseases, respiratory infectious diseases, natural foci and vector-borne infectious diseases, and STBBIs [13]. Due to the severity and data availability, this study focused on five STBBIs, including hepatitis B, hepatitis C, syphilis, gonorrhea and HIV infection, selected from the 27 Class B infectious diseases. Hepatitis D was excluded as complete data were only available from 2016 to 2021, and its diagnosis rates were very low. Although China has implemented network-based reporting for legally managed infectious diseases since 2004, the unstable infectious disease reporting data in 2004 prompted this study to only collect data on the five types of STBBIs nationwide from 2005 to 2021 across 31 provinces in China, except for Hong Kong, Macau, and Taiwan.

The data in this study were obtained from the Data-Center of China Public Health Science of National Population and Health Science Data Sharing Platform (https://www.phsciencedata.cn/Share/en/index.jsp) and the Chinese Center for Disease Control and Prevention (https://en.chinacdc.cn/). These two sources, both reporting data from the Chinese CDC, provided consistent information based on annual newly diagnosed cases. In this study, we directly collected data on the newly-reported cases and new diagnosis rates for each type of STBBI from 2005–2021 from the two sources. For each of the five STBBIs, we defined the yearly reported diagnosis rate as the number of newly diagnosed cases per capita in a given calendar year. Furthermore, we obtained the year-end population for each year between 2005 and 2021 from the China Statistical Yearbook, published by the National Bureau of Statistics (2022). Total yearly diagnosis rates for the five types of STBBIs from 2005–2021 were calculated by dividing the total number of annual newly diagnosed cases by the respective year-end population for each year.

### Statistical analyses

The joinpoint regression model [15], which comprises several continuous linear phases, was used to analyze the changes in STBBIs trends from 2005 to 2021. In this study, only log-linear joinpoint regression models were performed for analysis due to the non-normal distribution of STBBI diagnosis rate data, expressed as follows:

$${y}_{i}=ln{z}_{i}={\beta }_{0}+{\beta }_{1}{t}_{i}+{\gamma }_{1}{({t}_{i}-{\tau }_{1})}^{+}+\cdots \cdots +{\gamma }_{k}{({t}_{i}-{\tau }_{k})}^{+}+{\varepsilon }_{i}$$

where *i* = 1, 2, …., *n*, ${t}_{i}$ indicates the calendar year from 2005 to 2021 and ${z}_{i}$ represents the diagnosis rate of STBBIs. Here, ${\tau }_{k}$ (*k* = 1, 2, …, *K*) denotes the location of inflection point (joinpoint), with *K* indicating the number of inflection points, ${\beta }_{0}$, ${\beta }_{1}$ and ${\gamma }_{1}$, …, ${\gamma }_{k}$ denote the regression coefficients and ${\varepsilon }_{i}$ is the error term in the model. In addition, the notation ${\left({t}_{i}-{\tau }_{k}\right)}^{+}$ = ${t}_{i}-{\tau }_{k}$ if ${t}_{i}-{\tau }_{k}$ > 0, and ${({t}_{i}-{\tau }_{k})}^{+}$ = 0 otherwise. Monte Carlo permutation tests with 4499 randomly permuted data sets were used to determine the numbers and locations of the inflection points and to estimate the regression parameters [23], with the procedure for selecting the optimal joinpoint model being extensively discussed by Kim et al. (2000) [15]. Information on the default maximum number of joinpoints can be found on the Division of Cancer Control and Population Sciences (DCCPS) website (https://surveillance.cancer.gov/help/joinpoint/setting-parameters/method-and-parameters-tab/number-of-joinpoints). As our study covered a 17-year period, we specified a maximum of three joinpoints.

According to the fitted joinpoint regression model, the calculation formulas for the APC from year ${\tau }_{j}$(*j* = 1, 2, …, *J*) to year (${\tau }_{j}$ + 1) as follows:

$$APC = \left\{\mathrm{exp}\left({\beta }_{1}+{\gamma }_{1}+{\gamma }_{2}+\dots +{\gamma }_{j}\right)\right.-\left.1\right\}\times 100$$

Furthermore, for each fitted joinpoint regression model, the AAPC can be estimated as a weighted mean of the estimated APCs by using the segment lengths as weights.

In this study, APC is used to evaluate the internal trends of independent intervals of segmented functions or the overall trend with no joinpoints. AAPC is used to comprehensively evaluate the overall average trend of changes that include multiple intervals. If the inflection point is zero, then AAPC = APC. The APC > 0 means upward trend, APC < 0 means downward trend, and APC = 0 means stable state. The 95% confidence intervals for the APCs and AAPCs were calculated, and a t-test was used to perform hypothesis testing with a significance level of *p* < 0.05 [15]. We conducted the joinpoint regression analysis by using the Joinpoint Regression Program 4.9.1.0 (US National Cancer Institute, https://surveillance.cancer.gov/joinpoint/download).

## Results

### Diagnosis rate of all five STBBIs combined

**Fig. 1:**
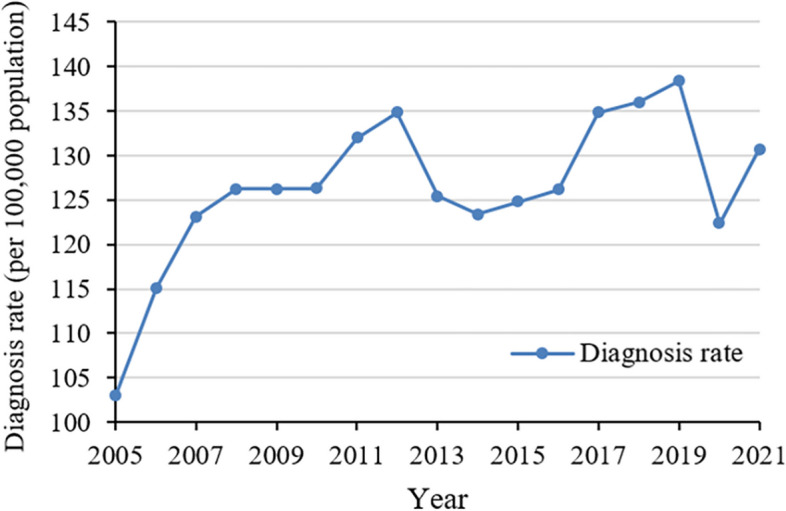
Annual diagnosis rate for five sexually transmitted and blood-borne infections in China, 2005–2021. Infections involved hepatitis B, hepatitis C, syphilis, gonorrhea or human immunodeficiency virus

From 2005 to 2021, a combined total of 29.4 million infections involving hepatitis B or C, syphilis, gonorrhea, or HIV were reported in China (Supplementary Table S1). Between 2005 and 2019, overall diagnosis rate of the five STBBIs increased continuously by 34.25% (Fig. 1, Supplementary Table S2). In 2020, the overall diagnosis rate dropped, but rebounded in 2021.

### Diagnosis rate of individual STBBIs

**Fig. 2:**
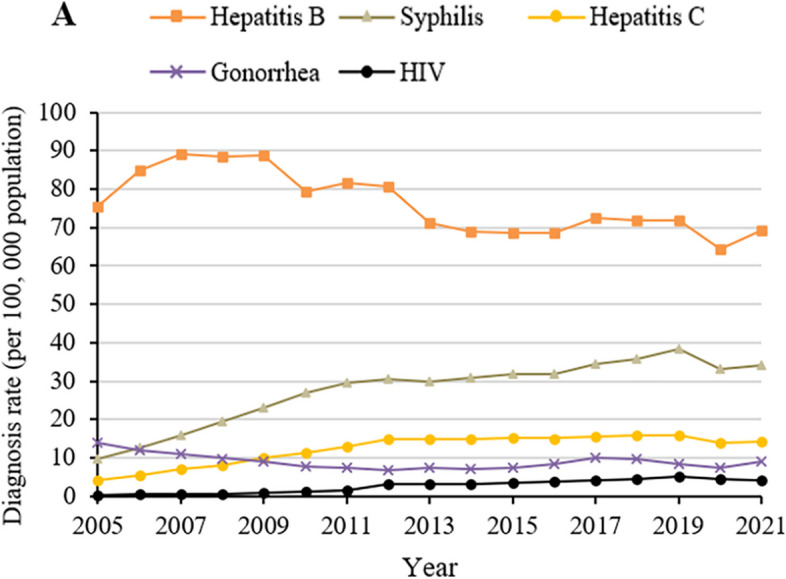
Annual diagnosis rate for five sexually transmitted and blood-borne infections in China, 2005–2021

Next, we analyzed diagnosis rate trends for each STBBI individually. During the period 2005–2021, diagnosis rate of infections with hepatitis B or gonorrhea decreased, whereas the diagnosis rate of infections with hepatitis C, HIV or syphilis increased (Fig. 2, Supplementary Table S3). The increase in HIV diagnosis rate was the most striking: it more than tripled during the period. These trends led to several changes in the relative diagnosis rate of the STBBIs during the 17-year period. While infections of hepatitis B ranked first and HIV ranked fifth throughout the period, syphilis moved up from third to second position, gonorrhea moved down from second to fourth position, and hepatitis C moved up from fourth to third position.

**Fig. 3:**
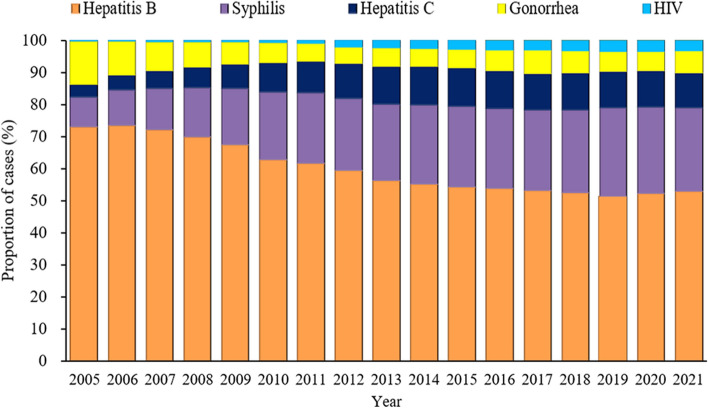
Proportions of all sexually transmitted and blood-borne infections in China, 2005 to 2021

Among all five STBBIs cases during the 17-year period, hepatitis B accounted for the largest proportion, followed by syphilis, hepatitis C, gonorrhea and HIV (Fig. 3 and Supplementary Table S4). During the period, the proportion of STBBIs due to hepatitis B fell from 72.89% to 52.86%, the proportion due to gonorrhea halved from 13.38% to 6.92%. Conversely, the proportion due to syphilis doubled from 9.38% to 25.99%, the proportion due to hepatitis C also doubled from 3.93% to 10.98%, and the proportion due to HIV increased eightfold from 0.42% to 3.26%.

### Inflection points (joinpoints) in diagnosis rate of all five STBBIs combined

**Fig. 4:**
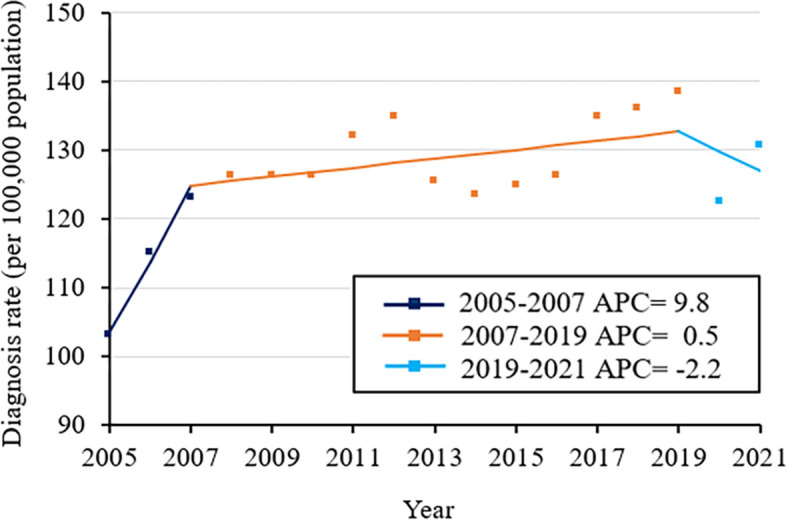
Trends in diagnosis rates of all five sexually transmitted and blood-borne infections together in China, 2005–2021. APC, annual percent change. * *p* < 0.05

Joinpoint regression analysis showed that the diagnosis rate of STBBIs in China increased from 2005 to 2021, with AAPC of 1.3% (95% CI -0.5% to 3.1%; Fig. 4 and Supplementary Table S5). The analysis identified three phases in the diagnosis rate trend: 2005–2007, when APC was 9.8%; 2007–2019, when APC was 0.5%; and 2019–2021, when APC was -2.2%.

### Inflection points (joinpoints) in diagnosis rate of individual STBBIs

**Fig. 5:**
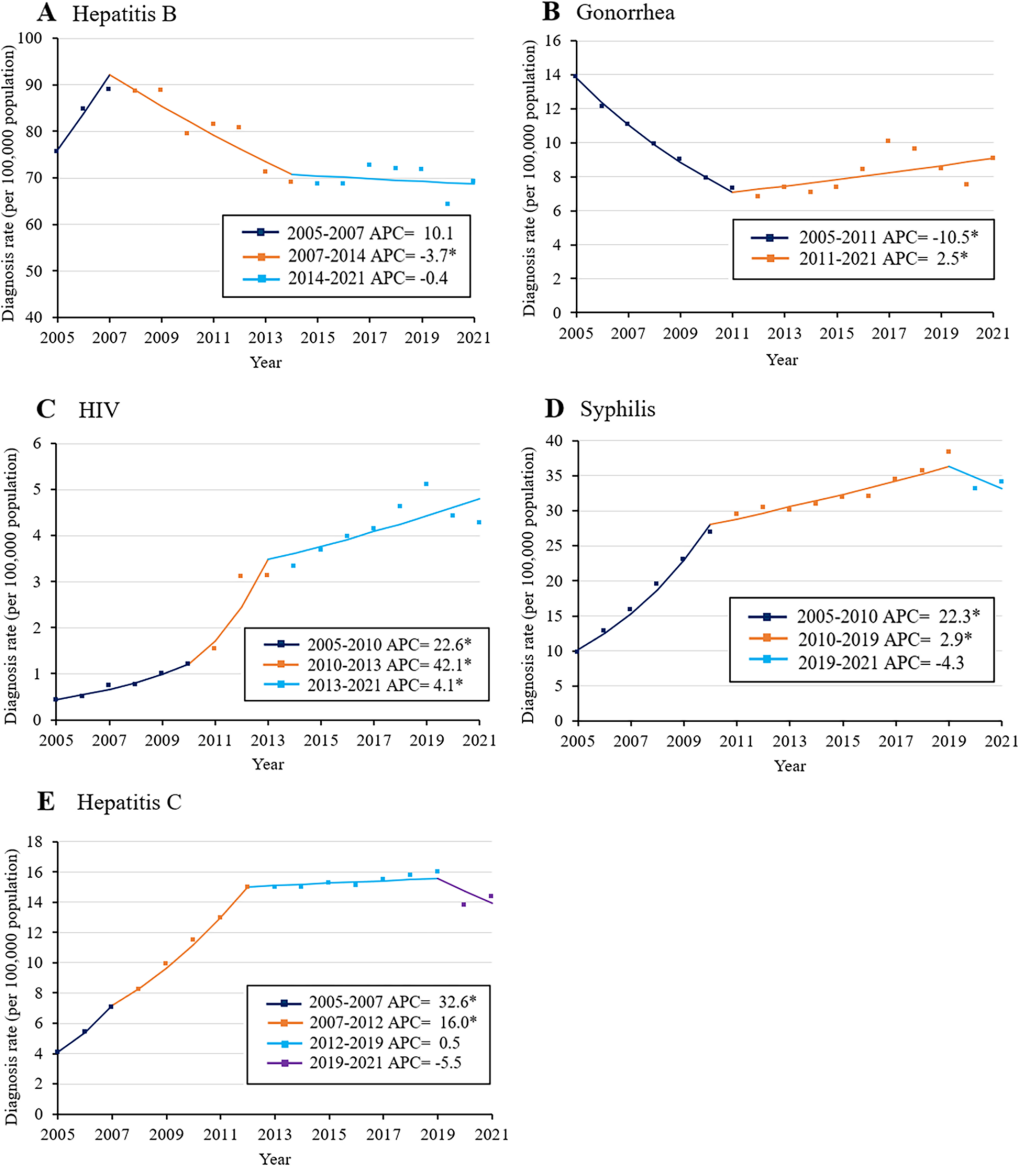
(**A**-**E**) Joinpoint analysis of trends in diagnosis rate of infections with (**A**) hepatitis B, (**B**) gonorrhea, (**C**) HIV, (**D**) syphilis, or (**E**) hepatitis C in China, 2005–2021. *p < 0.05

During the study period, the diagnosis rate of gonorrhea and hepatitis B showed a decreasing trend (Supplementary Table S6). Joinpoint regression identified three phases in the trend of diagnosis rate of hepatitis B: 2005–2007, 2007–2014, and 2014–2021 (Fig. 5A). The regression identified two phases in the diagnosis rate of gonorrhea infection, 2005–2011 and 2011–2021 (Fig. 5B).

Conversely, infections with HIV increased during the study period with an AAPC of 16.2%, and infections also increased in the case of hepatitis C (AAPC 8.0%) and syphilis (AAPC 7.6%). Joinpoint analysis identified three phases in the diagnosis rate of HIV: 2005–2010, when APC was 22.6%; 2010–2013, when APC was 42.1%; and 2013–2021, when APC was 4.1% (Fig. 5C). Similarly, it identified three phases in the case of syphilis: 2005–2010, when APC was 22.3%; 2010–2019, when APC was 2.9%; and 2019–2021, when the change in diagnosis rate was not significant (Fig. 5D). It identified four phases in the case of hepatitis C: 2005–2007, when APC was 32.6%; 2007–2012, when APC was 16%; 2012–2019, when APC was 0.5% but not significant; and 2019–2021, when APC was -5.5% but not significant (Fig. 5E).

## Discussion

This study showed that the overall diagnosis rate of all five STBBIs were on the rise in China from 2005 to 2021, although the rate of increase appears to be slowing. The overall diagnosis rate increased rapidly from 2005–2007 but leveled off after 2007. This suggests that although the prevention and control of STBBIs in China has made certain achievements in recent years, it remained an important public health challenge in China.

Several main factors may explain the increase in overall STBBI diagnosis rate during 2005–2019. First, the rapid economic and technological development in the country, together with the popularity of social media, have facilitated sexual encounters and a more open attitude towards sexuality in general, leading to increases in diagnosis rate of AIDS and gonorrhea [24]. Additionally, a series of studies in China have shown that most people, including college students, young men and men who have sex with men face many barriers to condom use and PrEP [25–27]. Another factor is the growing prevalence of STBBI screening.

The slowing down in the increase in STBBI diagnosis rate during 2019–2021 may be due, in part, to the COVID-19 pandemic. On the one hand, government efforts to limit the spread of the causative virus led to partial and total city lockdowns, restrictions on gathering and socializing, as well as traffic restrictions [28]. All these made sexual encounters more difficult. On the other hand, the observed decreases in annual newly diagnosed cases of STBBIs during the COVID-19 pandemic might also be attributed to reduced availability of diagnostic testing as individuals faced challenges accessing routine sexual healthcare and testing due to citywide lockdowns and travel restrictions. Indeed, diagnosis rate of the five STBBIs also declined in 2019–2021 [29], consistent with our observations.

Among the five STBBIs explored in this study, numbers of infections of hepatitis B and gonorrhea declined during the study period. Among these two, hepatitis B showed the highest diagnosis rate, which reflects the relatively high prevalence of this virus in China and widespread screening for it [30]. Indeed, the rapid increase in the diagnosis rate from 2005 to 2007 might be due to direct reporting of cases into a national infectious disease surveillance system since 2004 [31]. However, the diagnosis rate of hepatitis B declined faster than that of infection of the other four pathogens, possibly due to increasingly comprehensive strategies adopted by the Chinese government to prevent the spread of hepatitis B. In 2002, China incorporated the hepatitis B vaccine into the panel of required immunizations, resulting in an increase in hepatitis B vaccination rates [8]. Since 2008, hepatitis B has joined tuberculosis and HIV as three infectious diseases against which the Chinese government has prioritized its prevention and control efforts. In parallel, publicity campaigns have been launched and the healthcare sector has implemented comprehensive prevention and control programs, including measures to promote safe injection practices as well as systematic screening of blood donations [32].

These efforts, while effective, still have a long way to go, given that hepatitis B accounted for the greatest number and proportion of STBBIs during our 17-year study period. Indeed, one third of the 240 million people living with chronic hepatitis B worldwide reside in China [33, 34]. The diagnosis rate of hepatitis B has remained relatively constant since 2014, which is consistent with the findings of Zhang MY's study [20]. One reason may be that a large decrease as a result of widespread vaccination has already occurred and cannot be expected in the future. Another may be that government-funded programs offering free vaccination and testing services have operated in isolation from each other, preventing concerted efforts to control hepatitis B [35]. Better coordination of prevention efforts at the national level may further reduce the diagnosis rate of hepatitis B.

Gonorrhea diagnosis rate steadily decreased until 2011, at which point it slowly started to rise again. This trend is consistent with the findings of Wang YJ's study [18]. This could be attributed to multiple factors, including drug resistance acquired by *Neisseria* gonorrhea [36], increased national surveillance of sexually transmitted infections, more proactive testing, an increase in the number of people engaging in condomless sex, particularly male same-sex sexual behaviors as well as sex between female sex workers and their clients [37]. Policies to prevent gonorrhea may wish to take into account that incidence is higher in areas with high per capita gross domestic product, transient populations, inadequate health care, higher male–female ratio and high divorce rates [38].

The increase in diagnosis rate of HIV and syphilis during the study period may reflect an increase in sexual behavior, especially as people make use of social media to search for sexual partners [24]. HIV prevalence showed two inflection points in 2010 and 2013, and the rapid increase from 2010 to 2013 may be related not only to a large increase in infections among men having condomless male same-sex sexual behaviors, but also to increased rates of screening and detection among this group [39, 40]. Another potential explanation is the migration of people from high HIV-prone areas to big cities in order to achieve better living conditions and employment opportunities, which may have expanded the HIV epidemic [41].

Inflection points for syphilis diagnosis rate were detected in 2010 and 2019. From 2005 to 2010, the rapid increase in syphilis diagnosis rate could be accounted for by the considerable increase in congenital syphilis. Prior to 2010, health care efforts to prevent mother-to-child transmission focused on the prevention and control of HIV, and syphilis was not prioritized, possibly contributing to its inadequate prevention and control [42]. Syphilis can be co-transmitted with HIV, which may explain the observed rise in diagnosis rates of both types of infection [37]. In fact, the increased prevalence of co-screening for HIV and syphilis has led to higher detection of latent syphilis [43]. From 2010 to 2019, the rise in syphilis diagnosis rate slowed, which is similar to the findings of Ma N's study [19], and could possibly reflect new government policies [44, 45] to prevent and control syphilis, particularly mother-to-child transmission, in parallel with efforts to reduce vertical transmission of HIV and hepatitis B.

The diagnosis rate of hepatitis C showed an upward trend throughout the study period, but this leveled off after 2012, which is similar to the findings of Zhang MY's study [20]. This may reflect growing awareness of prevention, diagnosis, and treatment of hepatitis C among health professionals following the 2012 publication of the APASL Consensus Statements and Management Algorithms for Hepatitis C Virus Infection [46]. After 2019, the diagnosis rate of hepatitis C declined slightly, and this inflection point coincided with an inflection in the diagnosis rate of syphilis. It may be that the same government measures against COVID-19 reduced diagnosis rates of both types of infection. Another factor may be the release of a slate of government guidelines on how to screen vulnerable populations for hepatitis C and how to treat infection effectively [12, 47].

Our analysis leads to the following recommendations for future prevention and control of STBBIs in China. The general population should be educated about STBBIs, and anti-STBBI interventions should focus on vulnerable populations. Routine immunization and vaccination against hepatitis B should be expanded to all adults [48, 49]. Since syphilis and HIV share similar transmission routes and risk behaviors, prevention and control measures for both diseases should be tightly coordinated [37], mainly through screening of target populations and timely treatment of infected cases to curb transmission [50]. Strategies to reduce diagnosis rate of gonorrhea should focus on increasing condom accessibility to vulnerable populations, especially migrant workers and divorcees [38], and on developing alternative therapies to compensate for antibiotic resistance and lack of next-generation antibiotics in the pipeline [36]. Hepatitis C may be reduced through enhanced screening, strict screening of blood donors, prevention of sexual transmission and careful monitoring of potential mother-to-child transmission [35]. Additionally, measures to provide disposable needles and methadone substitution treatment for people who inject drugs should be continued, since needle sharing for drug use is the main transmission route of hepatitis C virus in China [51]. The eradication of STBBIs requires not only awareness and adoption of protective behaviors, but also the collaborative efforts of all sectors of society because STBBIs are closely related to socioeconomics. Finally, our analysis highlights the usefulness of regular assessments of trends in STBBIs in order to allow timely adjustment of strategies to maximize efficacy.

To our knowledge, this study is the first to use joinpoint regression models to evaluate the overall trends in STBBIs in China from 2005–2021. While the findings are of immediate relevance to researchers and policymakers, they should be interpreted carefully in light of some limitations. First, our data came from Chinese CDC reports, and a potential limitation of this study could be the impact on STBBIs trends due to changes in reporting practices and guidelines, as well as possible underreporting. Moreover, there were disparities in the STBBIs data between the CDC sources and the GBD and WHO sources. This disparity could potentially lead to inconsistent outcomes when comparing this study to those employing GBD and WHO sources. Nevertheless, we maintain faith in the Chinese CDC data's credibility as the most authoritative source in China, offering valuable insights for developing relevant policies and conducting research on STBBIs. Second, our study design is not causal inference, limiting the ability to determine which factors have caused the observed trends in STBBIs. Therefore, while this study discussed some possible factors for the trends in STBBIs, further research is needed to establish causal relationships. Third, our analysis was based on national STBBI data, which could be repeated in the future using province-level data to examine STBBI trends at greater resolution. Finally, we lacked data on STBBI diagnosis rate by age group, and were unable to standardize diagnosis rate.

## Conclusion

From 2005 to 2021, the overall diagnosis rate of STBBIs in China showed a continuous upward trend, but the overall growth rate slowed, suggesting that STBBI prevention and control in China has achieved some success. However, diagnosis rate of HIV, syphilis and hepatitis C continues to increase. Our results suggest the need to educate the general public about STBBIs and to target interventions to vulnerable groups. Finally, our analysis highlights the need for regular analyses of diagnosis rate trends in order to allow timely adjustment of public health policy.

### Supplementary Information

**Additional file 1. **

## Data Availability

The datasets supporting the conclusions of this article are publicly available from the Data-Center of China Public Health Science of National Population and Health Science Data Sharing Platform (https://www.phsciencedata.cn/Share/en/index.jsp) and the Chinese Center for Disease Control and Prevention (https://en.chinacdc.cn/).

## Abbreviations

APC: Annual percent change
AAPC: Average annual percent change
CI: Confidence interval
Chinese CDC: Chinese Center for Disease Control and Prevention
HIV: Human immunodeficiency virus
STBBIs: Sexually transmitted and blood-borne infections
AIDS: Acquired immunodeficiency syndrome
