# Supplementary material for: Trends in sexually transmitted and blood-borne infections in China from 2005 to 2021: a joinpoint regression model
Source: BMC Infect Dis. 2023 Oct 30;23:741. doi: 10.1186/s12879-023-08733-8 (PMC10614345; doi:10.1186/s12879-023-08733-8)
Supplement: Supplementary file 1 — Additional file 1. [file 12879_2023_8733_MOESM1_ESM.docx]

**SUPPLEMENTARY MATERIALS**

**Additional file 1: Table S1, Table S2,** **Table S3,** **Table S4,** **Table S5,** **Table S6.**

**Supplementary Table S1.** Annual newly-reported cases of all sexually transmitted and blood-borne infections in China that were due to each of the five pathogens in this study, 2005-2021

| **Year** | **Hepatitis B** | **Hepatitis C** | **Gonorrhea** | **Syphilis** | **HIV** | **Total** |  |
| --- | --- | --- | --- | --- | --- | --- | --- |
| 2005 | 982,297 | 52,927 | 180,316 | 126,445 | 5,621 | 1,347,606 | |
| 2006 | 1,109,130 | 70,681 | 158,795 | 167,370 | 6,671 | 1,512,647 | |
| 2007 | 1,169,946 | 92,378 | 145,597 | 208,784 | 9,727 | 1,626,432 | |
| 2008 | 1,169,569 | 108,446 | 130,818 | 257,474 | 10,059 | 1,676,366 | |
| 2009 | 1,179,607 | 131,849 | 119,824 | 306,381 | 13,281 | 1,750,942 | |
| 2010 | 1,060,582 | 153,039 | 105,544 | 358,534 | 15,982 | 1,693,681 | |
| 2011 | 1,093,335 | 173,872 | 97,954 | 395,182 | 20,450 | 1,780,793 | |
| 2012 | 1,087,086 | 201,622 | 91,853 | 410,074 | 41,929 | 1,832,564 | |
| 2013 | 962,974 | 203,155 | 99,659 | 406,772 | 42,286 | 1,714,846 | |
| 2014 | 935,702 | 202,803 | 95,473 | 419,091 | 45,145 | 1,698,214 | |
| 2015 | 934,215 | 207,897 | 100,245 | 433,974 | 50,330 | 1,726,661 | |
| 2016 | 942,268 | 206,832 | 115,024 | 438,199 | 54,360 | 1,756,683 | |
| 2017 | 1,001,952 | 214,023 | 138,855 | 475,860 | 57,194 | 1,887,884 | |
| 2018 | 999,985 | 219,375 | 133,156 | 494,867 | 64,170 | 1,911,553 | |
| 2019 | 1,002,292 | 223,660 | 117,938 | 535,819 | 71,204 | 1,950,913 | |
| 2020 | 902,476 | 194,066 | 105,160 | 464,435 | 62,167 | 1,728,304 | |
| 2021 | 976,233 | 202,771 | 127,803 | 480,020 | 60,154 | 1,846,981 | |
| Total | 17,509,649 | 2,859,396 | 2,064,014 | 6,379,281 | 630,730 | 29,443,070 | |

**Supplementary Table S2.** Annual overall diagnosis rate of all sexually transmitted and blood-borne infections in China that were due to each of the five pathogens in this study, 2005-2021

| **Year** | **Population** | **No. of cases** | **Diagnosis rate (per 100,000 population)** |
| --- | --- | --- | --- |
| 2005 | 130,765 | 1,347,606 | 103.0556 |
| 2006 | 131,448 | 1,512,647 | 115.0757 |
| 2007 | 132,129 | 1,626,432 | 123.0942 |
| 2008 | 132,802 | 1,676,366 | 126.2305 |
| 2009 | 133,450 | 1,750,942 | 131.2058 |
| 2010 | 134,091 | 1,693,681 | 126.3083 |
| 2011 | 134,916 | 1,780,793 | 131.9927 |
| 2012 | 135,922 | 1,832,564 | 134.8247 |
| 2013 | 136,726 | 1,714,846 | 125.4221 |
| 2014 | 137,646 | 1,698,214 | 123.3755 |
| 2015 | 138,326 | 1,726,661 | 124.8255 |
| 2016 | 139,232 | 1,756,683 | 126.1695 |
| 2017 | 140,011 | 1,887,884 | 134.8383 |
| 2018 | 140,541 | 1,911,553 | 136.0139 |
| 2019 | 141,008 | 1,950,913 | 138.3548 |
| 2020 | 141,212 | 1,728,304 | 122.3907 |
| 2021 | 141,260 | 1,846,981 | 130.7505 |
| Total | 2,321,485 | 29,443,070 | 126.8286 |

**Supplementary Table S3.** Average annual diagnosis rate of all sexually transmitted and blood-borne infections in China that were due to each of the five pathogens in this study, 2005-2021

| **Year** | **Hepatitis B** | **Hepatitis C** | **Gonorrhea** | **Syphilis** | **HIV** |
| --- | --- | --- | --- | --- | --- |
| 2005 | 75.5683 | 4.0717 | 13.8717 | 9.7274 | 0.4324 |
| 2006 | 84.8244 | 5.4056 | 12.1444 | 12.8002 | 0.5102 |
| 2007 | 89.0047 | 7.0277 | 11.0764 | 15.8834 | 0.7400 |
| 2008 | 88.5172 | 8.2076 | 9.9008 | 19.4866 | 0.7613 |
| 2009 | 88.8245 | 9.9282 | 9.0228 | 23.0705 | 1.0001 |
| 2010 | 79.4598 | 11.4658 | 7.9075 | 26.8617 | 1.1976 |
| 2011 | 81.5368 | 12.9667 | 7.3050 | 29.4712 | 1.5251 |
| 2012 | 80.6833 | 14.9643 | 6.8173 | 30.4356 | 3.1120 |
| 2013 | 71.1186 | 15.0036 | 7.3601 | 30.0414 | 3.1230 |
| 2014 | 69.0469 | 14.9651 | 7.0451 | 30.9254 | 3.3313 |
| 2015 | 68.5679 | 15.2589 | 7.3576 | 31.8521 | 3.6940 |
| 2016 | 68.7393 | 15.0886 | 8.3911 | 31.9670 | 3.9656 |
| 2017 | 72.6137 | 15.5107 | 10.0631 | 34.4867 | 4.1450 |
| 2018 | 71.9881 | 15.7926 | 9.5858 | 35.6251 | 4.6195 |
| 2019 | 71.7698 | 16.0153 | 8.4450 | 38.3677 | 5.0986 |
| 2020 | 64.2861 | 13.8239 | 7.4909 | 33.0831 | 4.4283 |
| 2021 | 69.2473 | 14.3832 | 9.0655 | 34.0493 | 4.2669 |

Values are per 100,000 population, unless otherwise specified.

**Supplementary Table S4.** Proportions of all sexually transmitted and blood-borne infections in China that were due to each of the five pathogens in this study, 2005-2021

| **Year** | **Hepatitis B** | **Hepatitis C** | **Gonorrhea** | | **Syphilis** | **HIV** | **Total** |
| --- | --- | --- | --- | --- | --- | --- | --- |
| 2005 | 72.89 | 3.93 | 13.38 | 9.38 | | 0.42 | 100.00 |
| 2006 | 73.32 | 4.67 | 10.50 | 11.06 | | 0.44 | 100.00 |
| 2007 | 71.93 | 5.68 | 8.95 | 12.84 | | 0.60 | 100.00 |
| 2008 | 69.77 | 6.47 | 7.80 | 15.36 | | 0.60 | 100.00 |
| 2009 | 67.37 | 7.53 | 6.84 | 17.50 | | 0.76 | 100.00 |
| 2010 | 62.62 | 9.04 | 6.23 | 21.17 | | 0.94 | 100.00 |
| 2011 | 61.40 | 9.76 | 5.50 | 22.19 | | 1.15 | 100.00 |
| 2012 | 59.32 | 11.00 | 5.01 | 22.38 | | 2.29 | 100.00 |
| 2013 | 56.16 | 11.85 | 5.81 | 23.72 | | 2.47 | 100.00 |
| 2014 | 55.10 | 11.94 | 5.62 | 24.68 | | 2.66 | 100.00 |
| 2015 | 54.11 | 12.04 | 5.81 | 25.13 | | 2.91 | 100.00 |
| 2016 | 53.64 | 11.77 | 6.55 | 24.94 | | 3.09 | 100.00 |
| 2017 | 53.07 | 11.34 | 7.36 | 25.21 | | 3.03 | 100.00 |
| 2018 | 52.31 | 11.48 | 6.97 | 25.89 | | 3.36 | 100.00 |
| 2019 | 51.38 | 11.46 | 6.05 | 27.47 | | 3.65 | 100.00 |
| 2020 | 52.22 | 11.23 | 6.08 | 26.87 | | 3.60 | 100.00 |
| 2021 | 52.86 | 10.98 | 6.92 | 25.99 | | 3.26 | 100.00 |

Values are %, unless otherwise specified.

**Supplementary Table S5.** Trends in diagnosis rate of sexually transmitted and blood-borne infections in China, 2005-2021.

| **Indicators** | **Duration** | **APC%**  **(95% CI)** | ***P*-value** | **AAPC%**  **(95% CI)** | ***P*-value** |
| --- | --- | --- | --- | --- | --- |
| **Diagnosis rate** | 2005-2007 | 9.8(-2.0, 22.9) | 0.095 | 1.3(-0.5, 3.1) | 0.167 |
|  | 2007-2019 | 0.5(-0.3, 1.3) | 0.165 |  |  |
|  | 2019-2021 | -2.2(-12.6, 9.5) | 0.668 |  |  |

APC, annual percent change; AAPC, average annual percent change. * *p* < 0.05

**Supplementary Table S6.** Trends in diagnosis rate of sexually transmitted and blood-borne infections stratified by disease type in China, 2005-2021.

| **Indicators** | **Disease** | **Duration** | **APC%**  **(95% CI)** | ***P-*value** | **AAPC%**  **(95% CI)** | ***P*-value** |
| --- | --- | --- | --- | --- | --- | --- |
| **diagnosis rate** | Hepatitis B | 2005-2007 | 10.1(-4.3, 26.7) | 0.156 | -0.6(-2.5, 1.3) | 0.512 |
|  |  | 2007-2014 | -3.7 (-6.0, -1.4) * | 0.006 |  |  |
|  |  | 2014-2021 | -0.4(-2.3, 1.5) | 0.626 |  |  |
|  | Gonorrhea | 2005-2011 | -10.5 (-14.7, -6.1) * | <0.001 | -2.6 (-4.5, -0.6) * | 0.012 |
|  |  | 2011-2021 | 2.5 (0.3, 4.8) * | 0.030 |  |  |
|  | HIV | 2005-2010 | 22.6 (15.1, 30.6) * | <0.001 |  |  |
|  |  | 2010-2013 | 42.1 (7.2, 88.2) * | 0.020 | 16.2 (10.4, 22.2) * | <0.001 |
|  |  | 2013-2021 | 4.1 (1, 7.4) * | 0.016 |  |  |
|  | Syphilis | 2005-2010 | 22.3 (19.9, 24.7) * | <0.001 | 7.6 (6.3, 8.9) * | <0.001 |
|  |  | 2010-2019 | 2.9 (1.9, 3.9) * | <0.001 |  |  |
|  |  | 2019-2021 | -4.3(-12.5, 4.5) | 0.284 |  |  |
|  | Hepatitis C | 2005-2007 | 32.6 (25.3, 40.4) * | <0.001 | 8.0 (6.9, 9.1) * | <0.001 |
|  |  | 2007-2012 | 16.0 (13.9, 18.1) * | <0.001 |  |  |
|  |  | 2012-2019 | 0.5(-0.4, 1.5) | 0.242 |  |  |
|  |  | 2019-2021 | -5.5(-10.7, 0.1) | 0.053 |  |  |

APC, annual percent change; AAPC, average annual percent change. * *p* < 0.05
